# Incidence and Risk Factors of Postoperative Bleeding After LLETZ

**DOI:** 10.3390/medsci14040478

**Published:** 2026-08-13

**Authors:** Chiara Paternostro, Elmar Joura, Gustav Nowotny, Eva Maria Langthaler, Frederic Toemboel, Sophie Pils

**Affiliations:** 1 Department of Obstetrics and Gynecology, Medical University of Vienna, Spitalgasse 23, 1090 Vienna, Austria; 2 Department of Pathology, Medical University of Vienna, Spitalgasse 23, 1090 Vienna, Austria; 3 Department of Anaesthesia, Intensive Care Medicine and Pain Medicine, Medical University of Vienna, Spitalgasse 23, 1090 Vienna, Austria

**Keywords:** LLETZ, postoperative bleeding, cervical dysplasia, cone length, anticoagulation, antiplatelet therapy, cervical infiltration, BMI

## Abstract

**Background:** Large loop excision of the transformation zone (LLETZ) is an established surgical procedure for cervical intraepithelial neoplasia; however, secondary postoperative bleeding may lead to unplanned outpatient visits, hospitalization, or surgical intervention. This study aimed to evaluate the incidence and risk factors of postoperative bleeding after LLETZ. **Methods:** We conducted a retrospective single-center cohort study comprising 1493 patients who underwent LLETZ for the treatment of cervical dysplasia between January 2008 and May 2023 at the Medical University of Vienna. Postoperative bleeding was defined as an unscheduled outpatient presentation for bleeding from the operative site within eight weeks after LLETZ requiring additional treatment. Clinical, surgical and histopathological parameters were analyzed. Univariable logistic regression was performed to identify factors associated with postoperative bleeding, with additional stratified analyses according to the use of intraoperative cervical infiltration. **Results:** Postoperative bleeding occurred in 56 patients (3.8%). The median time to bleeding was 13.5 days (IQR 9.0–17.0). Among patients with postoperative bleeding, 20 (35.7%) required hospitalization, 10 (50.0%) underwent operative intervention, and 2 (10.0%) required blood transfusion. In logistic regression, increasing cone length was associated with higher odds of postoperative bleeding (OR 1.83 per cm, 95% CI 1.06–3.18, *p* = 0.031). Anticoagulation and/or antiplatelet therapy was also associated with an increased bleeding risk (OR 3.61, 95% CI 1.46–8.96, *p* = 0.006). Higher BMI was associated with lower odds of postoperative bleeding (BMI per +5 kg/m^2^: OR 0.62, 95% CI 0.43–0.90, *p* = 0.011). **Conclusions:** LLETZ is associated with a low risk of clinically relevant postoperative bleeding, with an unplanned outpatient re-presentation rate of 3.8% and a re-operation rate of 0.7%. Increased cone length and anticoagulation and/or antiplatelet therapy were associated with higher bleeding rates. Larger prospective studies are needed to validate these findings and improve individualized perioperative risk assessment.

## 1. Introduction

Large loop excision of the transformation zone (LLETZ) is a well-established excisional treatment for high-grade cervical intraepithelial neoplasia (CIN) and has become the standard of care in most clinical settings [1,2]. Compared to ablative techniques such as laser ablation or cryotherapy, LLETZ enables accurate histopathological assessment in order to rule out invasive cancer and evaluates margin status precisely [3]. Furthermore, the risk of persistent or recurrent disease has been shown to be significantly higher with ablative techniques than with excisional procedures [4]. Additionally, LLETZ is a technically simple, cost-effective procedure that can also be performed in an outpatient setting under local anesthesia, enhancing its accessibility [3,5]. Alternative techniques for cervical conization include needle excision of the transformation zone, also termed straight wire excision of the transformation zone, as well as cold-knife and laser conization [6].

Postoperative adverse events of LLETZ include bleeding, infection or cervical stenosis. Secondary bleeding constitutes a clinically relevant complication, which can lead to unplanned outpatient visits, hospital readmissions or the need for emergency surgical intervention. It mostly occurs within 10–14 days after the surgery; however, published evidence defining the period of elevated risk for such complication is scarce [5]. The existing literature on the frequency of postoperative bleeding ranges from 0.4 to 36%, with most studies being retrospective [7,8,9,10]. The size of the excised cervical specimen appears to be a predictor of perioperative complications, particularly when the cone length exceeds 20 mm [8,11]. Other risk factors like intense postoperative exercise or the use of anticoagulation or antiplatelet therapy have been specified, although most of the available data often came from heterogeneous gynecological procedures rather than specifically from procedures on the cervix [11,12]. In general, findings across studies are inconsistent, and high-quality, procedure-specific evidence remains limited. The present study aimed to evaluate the incidence and risk factors of secondary postoperative bleeding following LLETZ in a large single-center cohort and place these findings in the context of the existing literature.

## 2. Material and Methods

### 2.1. Patient Population and Study Design

This retrospective, single-center cohort study included 1493 patients who underwent LLETZ for cervical dysplasia at the Department of Obstetrics and Gynecology, Medical University of Vienna, between January 2008 and May 2023.

Postoperative bleeding was defined as an unscheduled emergency presentation to the outpatient clinic due to bleeding from the operative site occurring between 24 h and eight weeks after LLETZ, requiring additional intervention, such as topical hemostatic agents, suturing, or electrocoagulation, in either an outpatient or inpatient setting. Bleeding events within the first 24 h were excluded, as patients generally remained hospitalized during this period. All postoperative bleeding events were clinically verified by a consultant during an outpatient examination. A gynecological speculum examination was performed to determine whether the bleeding originated from the wound bed or from another source. Intraoperative cervical infiltration was performed using 20 mL of 2% lidocaine with epinephrine 1:200,000, with 5 mL injected into each of the four cervical quadrants.

All patients underwent thorough preoperative assessment in the preanesthetic outpatient clinic, and medications such as antiplatelet and/or anticoagulation therapy were paused or adjusted according to the patients’ individual risk profiles, in line with clinical guideline recommendations and respective institutional protocols [12].

Histopathological findings were classified as negative (no evidence of dysplasia), low-grade squamous intraepithelial lesion (LSIL), high-grade squamous intraepithelial lesion (HSIL), adenocarcinoma in situ (AIS), squamous cell carcinoma (SCC), or adenocarcinoma (AC). The resection margin status of the LLETZ specimen was categorized as non-evaluable, negative, or positive. During the study period, all specimens were evaluated and graded by at least one of eight pathologists specialized in gynecological pathology.

The transformation zone (TZ) was classified as type 1 when the squamocolumnar junction was completely visible, type 2 when it was partially visible, and type 3 when it was not visible [13]. High-risk human papillomavirus (hr-HPV) genotyping was performed using the cobas^®^ HPV test (Roche Molecular Systems, Inc., Pleasanton, CA, USA). HPV 16 and HPV 18 were reported separately, whereas HPV genotypes 31, 33, 35, 39, 45, 51, 52, 56, 58, 59, 66, and 68 were collectively categorized as “Non-HPV 16/18 hr-HPV”.

### 2.2. Parameters Analyzed

Relevant data were collected retrospectively from the patients’ medical records. The main outcome parameter was incidence of postoperative bleeding within eight weeks after LLETZ. Furthermore, the following clinical characteristics were extracted: intraoperative cervical infiltration, use of anticoagulation or antiplatelet therapy, history of bleeding complication, smoking status, level of training of the operating surgeon (i.e., resident or consultant), dimension of the cone (length), TZ type, hr-HPV status, body mass index (BMI), age of the patient at LLETZ and histological result of the specimen including margin status.

### 2.3. Statistical Analysis

Nominal variables are presented as absolute numbers and frequencies, whereas continuous variables are summarized as medians with interquartile ranges (IQR). Statistical analyses were performed using IBM SPSS Statistics, version 31.0 (IBM Corp., Armonk, NY, USA). Between-group comparisons were conducted using the Mann–Whitney U test or Pearson’s chi-squared test, as appropriate. A univariable analysis was performed in order to evaluate the effects of intraoperative cervical infiltration, cone length, LLETZ performed by consultant or resident, use of anticoagulation and/or platelet therapy, history of bleeding complication, smoking and hr-HPV subtype on the occurrence of postoperative bleeding after LLETZ. Additional stratified analyses were conducted according to intraoperative infiltration status (no infiltration vs. infiltration) to evaluate a potential effect. Due to the limited number of outcome events, multivariable logistic regression including all potential predictors was not considered sufficiently robust. Therefore, univariable logistic regression analyses are presented as the primary analysis. Given the exploratory nature of the study, no adjustment for multiple comparisons was performed. Accordingly, individual *p*-values should be interpreted cautiously and considered hypothesis-generating. Because bleeding events were few overall (*n* = 56) and even fewer within subgroups (31 with cone-length data; 5 and 1 in the anticoagulation strata), the low events-per-variable ratio limits the precision of these estimates, which should also be interpreted as exploratory. Analyses were performed on a complete-case basis for each variable; therefore, denominators may differ between analyses according to data availability. No imputation of missing data was performed. A two-sided *p* value < 0.05 was considered significant. Table 1, Table 2 and Table 3 were generated by using Microsoft Word (version 16.89).

## 3. Results

A total of 1493 patients undergoing LLETZ were included in the analysis. Postoperative bleeding within 8 weeks after LLETZ requiring re-presentation to the outpatient clinic occurred in 56 patients (3.8%).

The median age was comparable between patients with and without postoperative bleeding (38 years, IQR 32–47, vs. 37 years, IQR 31–45, *p* = 0.563; Table 1). The cone length did not diverge significantly between groups (median 1.4 cm, IQR 1.0–2.0, in patients with bleeding vs. 1.3 cm, IQR 1.0–1.7, in patients without bleeding, *p* = 0.106). Postoperative bleeding occurred in 2.0% of patients with cone lengths ≤10 mm, 4.9% with lengths >10–15 mm, 2.9% with lengths >15–20 mm, and 5.8% with lengths >20 mm. Among patients with postoperative bleeding after LLETZ, the median time to bleeding was 13.5 days (IQR 9.0–17.0). Of these, 20 (35.7%) patients required hospitalization, of which 10 (50.0%) underwent operative intervention, and 2 (10.0%) required blood transfusion. Of the 10 patients who underwent operative intervention for postoperative bleeding, hemostasis was achieved with an absorbable hemostatic agent (oxidized regenerated cellulose) combined with diathermy in 7 cases, and with diathermy combined with suturing in 3 cases. Margin status was found to be similar between groups, with no significant differences for negative, positive, or non-evaluable margins. Likewise, the incidence of postoperative bleeding did not differ in patients operated by a consultant versus a resident (*p* = 0.638; Table 1).

Although all patients underwent surgery under general anesthesia, a total of 585 (39.2%) patients also received intraoperative cervical infiltration. This procedure was not associated with a statistically significant difference in postoperative bleeding rates (39.5% of patients without bleeding versus 32.1% of patients with bleeding, *p* = 0.271; Table 1). Table 2 shows that patients with and without intraoperative cervical infiltration had similar baseline characteristics. No statistically significant differences were observed regarding age, cone length, LLETZ performed by a resident or consultant, bleeding history, or use of anticoagulation and/or antiplatelet therapy. Postoperative bleeding rates and bleeding-related outcomes, including hospitalization, operative intervention, and blood transfusion, were also comparable between groups.

Patients with postoperative bleeding were more likely to have a history of bleeding (10.7% versus 6.8%), although this difference was not statistically significant (*p* = 0.267). In contrast, the use of anticoagulation and/or antiplatelet therapy was significantly more common among patients with postoperative bleeding compared with those without bleeding (10.7% vs. 3.2%, *p* = 0.003). In detail, 29 patients were receiving antiplatelet therapy, such as acetylsalicylic acid (ASA) or clopidogrel, one patient was on dual antiplatelet therapy (ASA and clopidogrel), one on fondaparinux, three were treated with low-molecular-weight heparin (LMWH), four with phenprocoumon, three with direct oral anticoagulants (DOACs) and two with a combination of a DOAC and antiplatelet therapy. Of the 43 patients receiving anticoagulation and/or antiplatelet therapy, six experienced postoperative bleeding (14.0%; two ASA, two LMWH, one fondaparinux, one phenprocoumon). The DOAC and phenprocoumon therapy were paused in accordance with current guideline recommendations and LMWH was last administered no less than 12 h preoperatively.

Among patients with available smoking data, smoking was less prevalent in those with postoperative bleeding than in those without postoperative bleeding (27.8% vs. 44.4%; *p* = 0.048).

The results of the univariable analysis evaluating clinical variables in relation to postoperative bleeding are presented in Table 3. Cone length data were available for 828 patients, including 797 patients without postoperative bleeding and 31 patients with postoperative bleeding. Median cone length was 1.4 cm (IQR 1.0–2.0) in patients with postoperative bleeding and 1.3 cm (IQR 1.0–1.7) in those without postoperative bleeding (*p* = 0.106). In univariable logistic regression, each 1 cm increase in cone length was associated with higher odds of postoperative bleeding (OR 1.83, 95% CI 1.06–3.18; *p* = 0.031). Moreover, the use of anticoagulation and/or antiplatelet therapy was also associated with a 3.6-fold higher odds ratio of postoperative bleeding (OR 3.61, 95% CI 1.46–8.96, *p* = 0.006). Smoking showed an inverse association that did not reach statistical significance (OR 0.48, 95% CI 0.23–1.01; *p* = 0.053), whereas higher BMI was associated with lower odds of postoperative bleeding (OR 0.62 per 5 kg/m^2^ increase, 95% CI 0.43–0.90; *p* = 0.011; Table 3).

A formal interaction term between anticoagulation/antiplatelet therapy and intraoperative cervical infiltration was fitted to test for effect modification. Among non-infiltrated patients (*n* = 908), 5 of 27 anticoagulated patients experienced postoperative bleeding versus 33 of 763 non-anticoagulated patients (OR 5.03, 95% CI 1.79–14.11); among infiltrated patients (*n* = 585), 1 of 16 anticoagulated patients bled versus 17 of 401 non-anticoagulated patients (OR 1.51, 95% CI 0.19–12.08). The interaction term was not statistically significant (Wald *p* = 0.31; likelihood-ratio *p* = 0.27), indicating that this difference in stratum-specific odds ratios could not be formally confirmed and may reflect chance given the small number of bleeding events among anticoagulated patients (5 and 1, respectively).

## 4. Discussion

This retrospective, single-center cohort study of 1493 patients who underwent LLETZ examined the incidence and risk factors for secondary bleeding. Bleeding occurred in 3.8% of cases within eight weeks after surgery.

This incidence is comparable with the findings of Kietpeerakool et al., who described a postoperative hemorrhage rate of 2.6% [7]. Other studies, such as Dunn et al. or Cokan et al., describe similar rates of 4.7% and 5.3%, respectively [9,14]. In contrast, Feddersen et al. reported a considerably higher incidence of postoperative vaginal bleeding following LLETZ, with up to 75.2% of women experiencing bleeding within the first few weeks after surgery [11]. A potential explanation for the divergent findings may lie in the active survey-based follow-up used in this study, which likely enabled the detection of mild bleeding events that would not have resulted in presentation to an outpatient clinic.

In the study cohort, median cone length did not differ significantly between women with versus without postoperative bleeding (1.4 cm [IQR 1.0–2.0] versus 1.3 cm [IQR 1.0–1.7], *p* = 0.106). In the logistic regression, cone length was significantly associated with postoperative bleeding with an odds ratio of 1.83 per cm increase (95% CI 1.06–3.18, *p* = 0.031). This finding is broadly consistent with the findings of Feddersen et al., who also reported a significant association between cone volume and postoperative bleeding, potentially attributed to a larger wound bed [11]. However, only cone length was consistently available in our cohort, whereas additional dimensions required to calculate cone volume were not recorded reliably throughout the study period. Direct comparison with their findings is therefore limited. Kietpeerakool et al. demonstrated that a LLETZ specimen length of 20 mm or more was significantly linked to postoperative complications, such as intra- and postoperative bleeding or infections, with an odds ratio of 2.09 (95% CI 1.39–3.14), which is comparable to our findings [7]. Although in this analysis cone-length distributions did not differ significantly in the non-parametric comparison, increasing cone length was associated with higher odds of postoperative bleeding in the continuous logistic-regression model. Given the limited number of bleeding events and the proportion of missing cone-length data, this association should be regarded as exploratory and requires confirmation in larger prospective studies.

In the study cohort, the incidence of secondary bleeding did not differ according to the surgeon’s level of training. Montanari et al. demonstrated that LLETZ specimens excised by residents have no significant difference in specimen length [15]. Several studies have investigated perioperative outcomes according to surgeon training level. However, most compare different gynecologic procedures rather than focusing specifically on LLETZ. For example, a meta-analysis by Bougie et al. reported a trend toward a higher risk of blood transfusion when residents perform surgery, whereas no such association was observed in this LLETZ-only study cohort [16].

A cervical block using lidocaine with epinephrine was performed by injection into all four quadrants of the cervix in order to reduce postoperative pain and provide vasoconstriction in 585 patients (39.2%). Postoperative bleeding did not differ significantly between patients who received a cervical block and those who did not (Table 1). A total of 43 of 1207 patients with available medication data (3.6%) were on anticoagulation and/or antiplatelet therapy, which was statistically significantly associated with an increased rate of postoperative bleeding (OR 3.61, 95% CI 1.46–8.96, *p* = 0.006; Table 3).

In the stratified analysis, the odds ratio for postoperative bleeding among anticoagulated/antiplatelet-treated patients was numerically higher among patients who did not receive cervical infiltration (OR 5.03) than among those who did (OR 1.51). However, a formal interaction term was not statistically significant (Wald *p* = 0.31; likelihood-ratio *p* = 0.27), so this apparent difference could not be confirmed and may simply reflect the very small number of bleeding events in each anticoagulated subgroup (5 and 1, respectively). This stratified comparison should therefore be regarded as exploratory and hypothesis-generating rather than evidence that cervical infiltration modifies anticoagulation-related bleeding risk.

Published data indicate that cervical block reduces primary bleeding due to its vasoconstrictive component but has less influence on secondary postoperative bleeding [17,18]. A meta-analysis of Martin-Hirsch et al. indicated that vasopressin reduces perioperative bleeding and was associated with a lower risk of bleeding requiring further sutures [19]. Due to the retrospective study design, assessment of postoperative pain scores was not possible. The literature suggests a beneficial effect of local cervical anesthesia, whether administered via intra- or paracervical block or by topical application of lidocaine spray [17,20].

According to the literature, antiplatelet therapy slightly increases the bleeding risk in gynecological surgery, especially by increasing the rate of primary bleeding without significantly augmenting bleeding severity or perioperative morbidity. ASA monotherapy increases the relative risk of procedural bleeding in gynecological surgeries by approximately 1.5-fold, whereas dual antiplatelet therapy (ASA plus clopidogrel) is associated with an absolute increase in major bleeding of 0.4–1.0% compared with ASA alone [21,22,23]. Moreover, patients receiving anticoagulant therapy who undergo gynecologic surgery have a two- to ten-fold higher bleeding risk than those not on anticoagulation, depending on the agent used, with direct oral anticoagulants associated with lower bleeding rates than warfarin [12,24,25,26,27]. Nonetheless, these data are generally derived from gynecologic surgeries of various types and are not specific to cervical procedures, underscoring the need for larger prospective studies targeting individual surgical interventions to improve patient safety and support individual counseling.

Smoking showed an inverse but statistically non-significant association with postoperative bleeding in the univariable analysis (OR 0.48, 95% CI 0.23–1.01; *p* = 0.053). Although smoking was less prevalent among patients with postoperative bleeding than among those without bleeding (27.8% vs. 44.4%; *p* = 0.048), this finding should be interpreted cautiously because smoking status was missing for 29.4% of the cohort, the number of bleeding events was limited, and the analysis was unadjusted. Accordingly, the finding should be regarded as exploratory and does not support a protective effect of smoking. Smoking is well-established as a factor that impairs wound healing and increases postoperative complications, and preoperative smoking cessation therefore remains recommended [28,29,30,31].

Another important risk factor for secondary bleeding could be the amount of exercise within the first weeks after the procedure, as Feddersen et al. described, particularly activities that place increased strain on the pelvis [11]. Nonetheless, there is limited evidence directly evaluating the impact of physical activity on postoperative bleeding risk in gynecologic procedures while moderate activity is generally considered safe. High-intensity or prolonged exercise in the early postoperative period is linked to an increased risk of postoperative bleeding [11,32]. In this retrospective analysis, postoperative lifestyle factors that may have contributed to an increased bleeding risk could not be assessed, underscoring the need for further prospective studies.

In our cohort, patients suffering from postoperative bleeding had a significantly lower BMI compared with those without. Although the absolute difference was modest, the median BMI was lower in the bleeding group than in the non-bleeding group (21.8 kg/m^2^ (IQR 20.8–24.2) vs. 23.2 kg/m^2^ (IQR 20.7–26.3), *p* = 0.002; Table 1). Consistently, logistic regression showed that each 5 kg/m^2^ increase in BMI was related to a lower odds of postoperative bleeding after LLETZ (OR 0.62, 95% CI 0.43–0.90, *p* = 0.011, Table 3). This finding suggests that lower BMI may be associated with an increased risk of clinically relevant postoperative bleeding. However, the clinical relevance of this association should be interpreted with caution, as BMI may also reflect other patient-related factors and the number of bleeding events was limited.

Strengths of this study are the large single-center cohort of 1493 patients undergoing LLETZ, providing a solid statistical power to evaluate incidence and risk factors of secondary bleeding. At our certified comprehensive cancer unit, all procedures were performed using a standardized surgical technique and were either conducted or supervised by a consultant specializing in gynecologic oncology.

Limitations include the retrospective study design, which precluded the assessment of potentially relevant factors such as postoperative physical or sexual activity and adherence to postoperative recommendations and resulted in missing data for several variables. Consequently, complete-case analyses were performed, which may have introduced selection bias. In addition, several patient- and procedure-related variables, including parity, menopausal status, hormonal contraception including the use of intrauterine devices (IUD) or hormone replacement therapy, menstrual-cycle phase at the time of surgery, and loop size, were either unavailable or not documented consistently.

Another possible limitation is that cases in which patients presented to outpatient clinics or hospitals other than our own may have been missed. Furthermore, although anticoagulation and antiplatelet therapy were identified as significant risk factors, the relatively small number of affected patients limited subgroup analyses according to specific agents or dosing regimens. Although cone length was available for analysis, additional dimensions recommended by the ESGO/EFC/IFCPC/ESP consensus, including specimen thickness, circumference, and volume, were not recorded consistently throughout the long study period. Their potential association with postoperative bleeding could therefore not be assessed.

A further limitation concerns generalizability across care settings. All procedures in this cohort were performed under general anesthesia in an inpatient surgical setting, whereas LLETZ is frequently performed as an outpatient procedure under local anesthesia. Anesthetic modality plausibly influences both the technique and extent of excision and the intraoperative hemostatic approach, and may also reflect differences in underlying patient selection between inpatient and outpatient practice. Bleeding rates and risk factors identified here may therefore not be directly generalizable to outpatient, local-anesthesia LLETZ, and comparisons with studies conducted in that setting should be interpreted with this difference in mind.

## 5. Conclusions

In conclusion, postoperative secondary bleeding after LLETZ occurred in a small proportion of patients and was comparable to other rates reported in the literature. Increased specimen length and the use of anticoagulant and/or antiplatelet therapy were significantly associated with postoperative bleeding, whereas surgeon experience and the use of cervical application of a local anesthetic combined with a vasoconstrictive agent did not significantly influence secondary bleeding rates. In summary, LLETZ is associated with a low risk of clinically relevant postoperative bleeding, with an unplanned outpatient re-presentation rate of 3.8% and a re-operation rate of 0.7%.

Overall, these findings highlight the multifactorial nature of secondary bleeding after LLETZ and emphasize the need for larger prospective, procedure-specific studies to better inform perioperative management, postoperative counseling, and individualized risk assessment.

## Figures and Tables

**Table 1:** Baseline Characteristics and Postoperative Bleeding Outcomes in the Overall Cohort.

Variable	Overall Cohort (*n* = 1493)	No Postoperative Bleeding (*n* = 1437)	Postoperative Bleeding (*n* = 56)	*p*-Value
Age, years	37 (31–45)	37 (31–45)	38 (32–47)	0.563
Negative margins	1055 (70.7)	1018 (70.8)	37 (66.1)	0.435
Positive margins	412 (27.6)	394 (27.4)	18 (32.1)	0.435
Non-evaluable margin status	26 (1.7)	25 (1.7)	1 (1.8)	—
Previous LLETZ	107/990 (10.8)	105/951 (11.0)	2/39 (5.1)	0.244
Cone length, cm	1.3 (1.0–1.7)	1.3 (1.0–1.7)	1.4 (1.0–2.0)	0.106
Days from LLETZ to bleeding	—	—	13.5 (9.0–17.0)	—
LLETZ performed by consultant	632 (42.3)	610 (42.4)	22 (39.3)	0.638
LLETZ performed by resident	861 (57.7)	827 (57.6)	34 (60.7)	—
Intraoperative cervical infiltration	585 (39.2)	567 (39.5)	18 (32.1)	0.271
Postoperative bleeding	56 (3.8)	—	—	—
Hospitalization due to bleeding	20/56 (35.7)	—	—	—
Operative intervention due to bleeding	10/20 (50.0)	—	—	—
Red blood cell transfusion due to bleeding	2/20 (10.0)	—	—	—
Positive bleeding history	81/1154 (7.0)	75/1098 (6.8)	6/56 (10.7)	0.267
Anticoagulant and/or antiplatelet therapy	43/1207 (3.6)	37/1151 (3.2)	6/56 (10.7)	0.003
BMI, kg/m^2^	23.1 (20.7–26.2)	23.2 (20.7–26.3)	21.8 (20.8–24.2)	0.002
Smoking	462/1054 (43.8)	452/1018 (44.4)	10/36 (27.8)	0.048
HPV 16	510/1088 (46.9)	492/1049 (46.9)	18/39 (46.2)	0.637
HPV 18	98/1088 (9.0)	93/1049 (8.9)	5/39 (12.8)	0.474
Non-HPV 16/18 hr-HPV	480/1088 (44.1)	464/1049 (44.2)	16/39 (41.0)	0.444
Transformation zone type 1	780/1345 (58.0)	757/1302 (58.1)	23/43 (53.5)	0.572
Transformation zone type 2	305/1345 (22.7)	293/1302 (22.5)	12/43 (27.9)	0.480
Transformation zone type 3	260/1345 (19.3)	252/1302 (19.4)	8/43 (18.6)	0.958
Histological diagnosis: negative	103 (6.9)	99 (6.9)	4 (7.1)	0.941
Histological diagnosis: LSIL	156 (10.4)	152 (10.6)	4 (7.1)	0.410
Histological diagnosis: HSIL	1113 (74.5)	1072 (74.6)	41 (73.2)	0.815
Histological diagnosis: AIS	41 (2.7)	39 (2.7)	2 (3.6)	0.700
Histological diagnosis: SCC	67 (4.5)	62 (4.3)	5 (8.9)	0.102
Histological diagnosis: AC	13 (0.9)	13 (0.9)	0 (0.0)	0.475

Data are presented as median (interquartile range) or n/N (%), unless otherwise indicated. For variables with complete data, percentages are based on the respective column total (overall cohort, *n* = 1493; no postoperative bleeding, *n* = 1437; postoperative bleeding, *n* = 56). For variables with missing data, the available denominator is shown separately in each column as n/N, and percentages are calculated using that denominator. Abbreviation: LLETZ, large loop excision of the transformation zone.

**Table 2:** Baseline Characteristics and Bleeding Outcomes According to Intraoperative Cervical Infiltration.

Variable	No Infiltration (*n* = 908)	Infiltration (*n* = 585)	*p*-Value
Age, years	36.0 (31.0–45.0)	37.0 (31.0–45.0)	0.564
Cone length, cm	1.3 (1.0–1.7)	1.3 (1.0–1.7)	0.753
LLETZ performed by consultant	394 (43.4)	238 (40.7)	0.327
Positive bleeding history	55/743 (7.4)	26/411 (6.3)	0.572
Anticoagulant and/or antiplatelet therapy	27/790 (3.4)	16/417 (3.8)	0.833
Postoperative bleeding	38 (4.2)	18 (3.1)	0.271
Hospitalization due to bleeding	14/38 (36.8)	6/18 (33.3)	1.000
Operative intervention due to bleeding (*n* = 20/56)	8/38 (21.1)	2/18 (11.1)	0.474
Red blood cell transfusion due to bleeding (*n* = 2/56)	2/38 (5.3)	0/18 (0.0)	1.000

Data are presented as median (interquartile range) or n (%), unless otherwise indicated. Percentages are based on the respective infiltration-group total unless an explicit n/N denominator is shown. Abbreviation: LLETZ, large loop excision of the transformation zone.

**Table 3:** Univariable Analysis of Potential Associated Factors for Postoperative Bleeding.

Variable	OR	95% CI	*p*-Value
Intraoperative cervical infiltration	0.73	0.41–1.29	0.273
Cone length, cm	1.83	1.06–3.18	**0.031**
LLETZ performed by consultant	0.88	0.51–1.51	0.638
Positive bleeding history	1.64	0.68–3.94	0.272
Anticoagulant and/or antiplatelet therapy	3.61	1.46–8.96	**0.006**
Smoking	0.48	0.23–1.01	0.053
HPV 16	0.89	0.47–1.71	0.738
HPV 18	1.30	0.49–3.41	0.596
Non-HPV 16/18 hr-HPV	0.86	0.46–1.64	0.656
BMI per 5 kg/m^2^ increase	0.62	0.43–0.90	**0.011**

Abbreviations: BMI, body mass index; CI, confidence interval; HPV, human papillomavirus; hr-HPV, high-risk human papillomavirus; LLETZ, large loop excision of the transformation zone; OR, odds ratio.

## Data Availability

The data presented in this study are available from the corresponding author upon reasonable request. The data are not publicly available due to institutional and data protection considerations.

## Abbreviations

The following abbreviations are used in this manuscript:

AC	adenocarcinoma
AI	artificial intelligence
AIS	adenocarcinoma in situ
ASA	acetylsalicylic acid
BMI	body mass index
CI	confidence interval
CIN	cervical intraepithelial neoplasia
DOAC	direct oral anticoagulant
HPV	human papillomavirus
hr-HPV	high-risk human papillomavirus
HSIL	high-grade squamous intraepithelial lesion
IQR	interquartile range
LLETZ	large loop excision of the transformation zone
LMWH	low-molecular-weight heparin
LSIL	low-grade squamous intraepithelial lesion
OR	odds ratio
SCC	squamous cell carcinoma
SPSS	Statistical Package for the Social Sciences
TZ	transformation zone
